# Mystery Shopper Study of State Medicaid Coverage for Out-of-State Abortion Care

**DOI:** 10.1001/jamanetworkopen.2023.43569

**Published:** 2023-11-15

**Authors:** Hazar Khidir, Carlisle Topping, Vanessa K. Dalton, Stacy Tessler Lindau, Arjun K. Venkatesh

**Affiliations:** 1 National Clinician Scholars Program, Yale University School of Medicine, New Haven, Connecticut; 2 Yale University School of Medicine, New Haven, Connecticut; 3 Department of Obstetrics and Gynecology, University of Michigan, Ann Arbor; 4 Department of Obstetrics and Gynecology, University of Chicago, Chicago, Illinois; 5 Department of Medicine-Geriatrics and Palliative Medicine, University of Chicago, Chicago, Illinois; 6 Department of Emergency Medicine, Yale University School of Medicine, New Haven, Connecticut

## Abstract

This cross-sectional study uses a mystery shopper survey to examine variation in state Medicaid agency responses to coverage questions on out-of-state abortion care.

## Introduction

The Supreme Court *Dobbs v Jackson Women’s Health Organization* decision has exacerbated state-level variability in abortion access.^1^ Some pregnant people have turned to abortion care out of state.

Approximately 35% of people seeking abortion care rely on Medicaid.^2^ The Hyde Amendment prohibits use of federal funds for abortion except in cases of life endangerment, rape, or incest, although some states use state funding to cover in-state abortions beyond these exceptions.^3,4^ Federal regulation requires state Medicaid plans to cover out-of-state services in medical emergencies.^5^ Little is known about whether state Medicaid agencies cover abortion care obtained out of state. This cross-sectional study used a mystery shopper survey to examine variation in state Medicaid agency responses to coverage questions on out-of-state abortion care.

## Methods

The Yale University institutional review board deemed this cross-sectional study nonhuman participants research, so informed consent was not required. The STROBE reporting guideline was followed.

Between August 22 and September 9, 2022, 3 female callers surveyed Medicaid agencies in all 50 states and the District of Columbia using a mystery shopper design (eAppendix in Supplement 1). Using a standardized script, callers stated they were calling on behalf of an adult beneficiary seeking out-of-state abortion care. Callers asked about out-of-state abortion coverage in general and in a medical emergency. Callers did not inquire about coverage for other exceptions but documented when they were mentioned by representatives. Callers spoke with the first available representative. A standard form was used to document responses and descriptive statistics to summarize responses. A summary of in-state abortion laws and Medicaid abortion coverage policies is provided in the eTable in Supplement 1.

## Results

A total of 122 calls were made to 51 agencies; 40 (78%) responded to questions about coverage for out-of-state abortion, 7 (14%) were unreachable, and 4 (8%) could not be surveyed. Of 40 representatives surveyed, 4 (10%) did not know if out-of-state abortion was covered, 4 (10%) said out-of-state abortion was not covered in a medical emergency, 32 (80%) said out-of-state abortion for a medical emergency would be covered; of these, 8 indicated it was also covered in other circumstances (Table 1).

**Table 1.** Mystery Shopper Survey Responses: Medicaid Coverage for Out-of-State Abortion^a^

Medicaid agency	Mystery shopper survey response: Medicaid coverage for out-of-state abortion
Alabama Medicaid	Did not know
Alaska Medicaid	Not surveyed
Arizona Medicaid	Not surveyed
Arkansas Medicaid	Medical emergency covered^b^
California Medicaid	Medical emergency covered^b^
Colorado Medicaid	Medical emergency covered
Connecticut Medicaid	Medical emergency covered
Delaware Medicaid	Medical emergency covered
District of Columbia Medicaid	Not surveyed
Florida Medicaid	Not surveyed
Georgia Medicaid	Coverage beyond medical emergency^c^
Hawaii Medicaid^d^	Medical emergency covered
Idaho Medicaid	Did not know
Illinois Medicaid	Not surveyed
Indiana Medicaid	Medical emergency covered
Iowa Medicaid	Not surveyed
Kansas Medicaid^d^	Medical emergency covered
Kentucky Medicaid	Did not know
Louisiana Medicaid	Not covered in medical emergency
Maine Medicaid	Medical emergency covered
Maryland Medicaid	Not surveyed
Massachusetts Medicaid	Not surveyed
Michigan Medicaid	Coverage beyond medical emergency^c^
Minnesota Medicaid	Coverage beyond medical emergency^e^
Mississippi Medicaid	Not covered in medical emergency^e^
Missouri Medicaid	Not surveyed
Montana Medicaid	Coverage beyond medical emergency^f^
Nebraska Medicaid	Medical emergency covered
Nevada Medicaid^d^	Medical emergency covered
New Hampshire Medicaid^d^	Medical emergency covered
New Jersey Medicaid	Medical emergency covered
New Mexico Medicaid	Medical emergency covered
New York Medicaid	Medical emergency covered
North Carolina Medicaid	Not covered in medical emergency
North Dakota Medicaid	Coverage beyond medical emergency^e^
Ohio Medicaid	Medical emergency covered
Oklahoma Medicaid	Medical emergency covered
Oregon Medicaid^d^	Medical emergency covered
Pennsylvania Medicaid	Medical emergency covered^b^
Rhode Island Medicaid^d^	Coverage beyond medical emergency^e^
South Carolina Medicaid	Not surveyed
South Dakota Medicaid	Not surveyed
Tennessee Medicaid	Did not know
Texas Medicaid	Coverage beyond medical emergency^e^
Utah Medicaid	Medical emergency covered
Vermont Medicaid	Coverage beyond medical emergency^e^
Virginia Medicaid	Not covered in medical emergency
Washington Medicaid^d^	Medical emergency covered
West Virginia Medicaid^d^	Medical emergency covered
Wisconsin Medicaid	Medical emergency covered
Wyoming Medicaid	Medical emergency covered

^a^ One Medicaid representative surveyed per agency.

^b^ Agency representative did not know if abortion is covered beyond cases of medical emergency.

^c^ Agency representative indicated coverage also available for nonmedical emergency cases that receive approval through prior authorization.

^d^ Central Medicaid representative referred caller to Medicaid managed care organization, which was surveyed.

^e^ Agency representative indicated coverage available for rape and incest.

^f^ Agency representative indicated out-of-state abortion generally covered if out-of-state provider accepts Montana Medicaid.

Of 32 representatives reporting coverage for medical emergencies, 20 defined medical emergency as a life-threatening circumstance (Table 2). Representatives indicated that a variety of entities decided whether the definition of a medical emergency was met. Twelve representatives said out-of-state abortion may require prior authorization. Only 9 representatives responded with certainty that both medical and surgical abortion care would be covered.

**Table 2.** Mystery Shopper Responses: Coverage Criteria for Out-of-State Abortion in a Medical Emergency

Information queried	No. (%) of state Medicaid agencies (N = 32)^a^
What is considered a medical emergency	
Life endangerment	20 (63)
Danger to physical or emotional health	1 (3)
Medical emergency (not otherwise specified)	7 (22)
Medical emergency (provided example: accident, miscarriage)	3 (9)
Did not know	1 (3)
Entity determining whether case is a medical emergency^b^	
Emergency physician or hospital	13 (41)
Per insurance retrospective review of medical records	6 (19)
Beneficiary’s primary care physician	2 (6)
Nurse hotline	3 (9)
Medical director or physician committee at agency	2 (6)
Not specified	7 (22)
Did not know	2 (6)
Administrative requirements for out-of-state abortion coverage^c^	
Emergency clinician documents medical emergency	12 (38)
Out-of-state clinician registered at home-state Medicaid agency	11 (34)
Prior authorization form	12 (38)
Submitted by out-of-state clinician: to provide abortion services	11 (34)
Submitted by primary care clinician: to see out-of-network clinician	2 (6)
Verification that health service is not available in state	1 (3)
Consent form	1 (3)
Did not know	5 (16)
Type of abortion services covered	
Both medical and procedural abortion covered	8 (25)
Procedural abortion only (pill not covered)	1 (3)
Did not know	23 (72)

^a^ Denominator is agencies that reported out-of-state abortion coverage in a medical emergency.

^b^ Some agencies specified more than 1 entity helps determine whether case is considered a medical emergency.

^c^ Some agencies specified that more than 1 administrative requirement would be necessary.

## Discussion

We found variability in agency staff representation of coverage policies for out-of-state abortion care. Some representatives provided information inconsistent with federal policy (eg, 4 state Medicaid agency representatives indicated that Medicaid would not cover out-of-state abortion in a medical emergency). Two of these states, Mississippi and Louisiana, had near-total abortion bans in effect at time of data collection. Most representatives (80%) told callers that abortion care rendered out-of-state would be covered only in a medical emergency. This highlights the need for states to establish Medicaid waivers for pregnant people traveling across state lines to seek abortion care.^6^

Even in a medical emergency, coverage barriers exist, primarily lack of a consistent, pragmatic definition of medical emergency that can be implemented in real time. Most agencies defined medical emergency as “life endangerment.” Yet, agencies commonly indicated that determination of life endangerment was made retrospectively by various entities, some without expertise in emergency care. Furthermore, some states required emergency physicians to submit prior authorization. Delayed or retrospective application of a life-endangerment standard threatens the lives and livelihoods of pregnant people and creates legal risk and moral distress for clinicians. Federal policy should establish common administrative requirements for out-of-state abortion coverage and monitor states’ abortion claims decisions to ensure adherence to federal regulations. Our study was limited in that findings were based on 1 round of calls to each state agency, so we could not assess for consistency in coverage information within states.

## Notes

Supplement 1. **eAppendix.** Mystery Shopper Study Rationale and Methodology
eReferences.
**eTable.** (1) Legal Status of Abortion and (2) State Medicaid Abortion Coverage Policies in Each State or District in the United States at the Time of Data Collection

Supplement 2.
Data Sharing Statement

## References

[1] Deal L. State laws restricting or prohibiting abortion. Congressional Research Service. 2023. Accessed August 27, 2023. https://crsreports.congress.gov/product/pdf/LSB/LSB10779

[2] Jerman J, Jones RK, Onda T. Characteristics of U.S. abortion patients in 2014 and changes since 2008. Guttmacher Institute. Published April 21, 2016. Accessed September 9, 2022. https://www.guttmacher.org/report/characteristics-us-abortion-patients-2014

[3] Guttmacher Institute. State funding of abortion under Medicaid. Published August 2022. Accessed August 30, 2023. https://web.archive.org/web/20220803195047/https://www.guttmacher.org/state-policy/explore/state-funding-abortion-under-medicaid

[4] Dennis A, Blanchard K. A mystery caller evaluation of Medicaid staff responses about state coverage of abortion care. Womens Health Issues. 2012;22(2):e143-e148. doi:10.1016/j.whi.2011.11.001 22154890

[5] GovInfo. 42 CFR 431.52—payments for services furnished out of state. Accessed September 8, 2022. https://www.govinfo.gov/app/details/CFR-2012-title42-vol4/CFR-2012-title42-vol4-sec431-52

[6] U.S. Department of Health and Human Services. HHS takes action to strengthen access to reproductive health care, including abortion care. Published August 26, 2022. Accessed October 8, 2022. https://www.hhs.gov/about/news/2022/08/26/hhs-takes-action-strengthen-access-reproductive-health-care-including-abortion-care.html

