# Supplementary material for: Mystery Shopper Study of State Medicaid Coverage for Out-of-State Abortion Care
Source: JAMA Netw Open. 2023 Nov 15;6(11):e2343569. doi: 10.1001/jamanetworkopen.2023.43569 (PMC10652153; doi:10.1001/jamanetworkopen.2023.43569)
Supplement: Supplement 1. — eAppendix. Mystery Shopper Study Rationale and Methodology eReferences. eTable. (1) Legal Status of Abortion and (2) State Medicaid Abortion Coverage Policies in Each State or District in the United States at the Time of Data Collection [file jamanetwopen-e2343569-s001.pdf]

Supplementary Online Content

Khidir H, Topping C, Dalton VK, Lindau ST, Venkatesh AK. Mystery shopper study of state Medicaid coverage for out-of-state abortion care. *JAMA Netw Open.* 2023;6(11):e2343569. doi:10.1001/jamanetworkopen.2023.43569

eAppendix. Mystery Shopper Study Rationale and Methodology
eReferences.
eTable. (1) Legal Status of Abortion and (2) State Medicaid Abortion Coverage Policies in Each State or District in the United States at the Time of Data Collection

This supplementary material has been provided by the authors to give readers additional information about their work.

© 2023 Khidir H et al. *JAMA Network Open.*

eAppendix. Mystery Shopper Study Rationale and Methodology

Study Design
We conducted a cross-sectional telephone survey of state Medicaid customer service hotlines of all 50 states and the district of Columbia. We used a mystery shopper approach because, at the time of study design and data collection, there was an absence of written policy data on out-of-state Medicaid coverage specifically for abortion across most states. Additionally, *de jure* policies related to abortion coverage have been found to differ from *de facto* abortion coverage practices; studies have found that Medicaid coverage for in-state abortions is inaccessible even in cases that should qualify for coverage.[1–3] The mystery shopper design sought to approximate the practical experience of a beneficiary's access to information on out-of-state abortion coverage.

Study Sample
We generated a list of publicly available Medicaid customer service numbers. We specifically called member/beneficiary benefits support lines when this direct phone number was publicly available on state Medicaid websites. We spoke to the first customer service agent willing to answer questions. Some staff members transferred or referred callers to other Medicaid service lines. Five of the state agencies survey responses resulted from calls that were transferred to other lines within Medicaid or the Department of Health and Human Services. Eight of the survey responses (Hawaii, Kansas, Nevada, New Hampshire, Oregon, Rhode Island, Washington, West Virginia) resulted from referrals to Medicaid Managed Care phone lines from central Medicaid helplines. Given that Medicaid hotlines are an important resource for beneficiaries, we terminated call attempts when hold times were anticipated to exceeded 10 minutes. At least 3 call attempts were made before an agency was deemed unreachable.

Data Collection
Medicaid staff and answering services request that callers provide a Medicaid beneficiary identification number or social security number at the start of customer service calls. Given that we were unable to provide requested information, callers posed as the sister of an adult Medicaid beneficiary seeking out-of-state abortion services. Callers indicated they were calling on behalf of a family member who was beneficiary, was visiting from out-of-state, and was seeking out-of-state abortion services.

Using a standardized script, callers first queried whether abortion would be covered generally out of state ("I was wondering if there's any way that the abortion would be covered by her ___[State Medicaid Agency]_____ if she does it out-of-state?"). Callers also inquired about coverage for a medical emergency ("What if it's an emergency?") if staff members answered 'no' or did not mention that abortion would be covered in a medical emergency in their response. Of note, callers did not explicitly ask about exceptions beyond medical emergency (rape, incest, etc) though these were often mentioned by staff, and we documented when staff mentioned them.

Callers also inquired about criteria for emergency abortion ("What criteria does she have to meet for it to be considered a medical emergency?"). Callers also inquired about whether pill and/or surgical abortion would be covered, the process for getting the abortion covered and whether any special paperwork or information would need to be provided by the beneficiary or doctor. We were unable to record calls. Callers instead used a standardized data collection form to collect notes during and after the call. We did not document any identifying information about agency representatives.
Data on in-state abortion restrictions [Appendix B, Column (1)] was obtained using two primary web sources, the Guttmacher Institute and the Center for Reproductive Rights.[4,5] We used a web archive source to look back at the Guttmacher Institute and Center for Reproductive Rights state legislative

© 2023 Khidir H et al. *JAMA Network Open.*

trackers websites on the date the mystery shopper call was conducted for each state. When there was inconsistency in the legal policies reported on the two web sources, we adjudicated the legal status of abortion at the time of data collection using a third source. Data on state Medicaid abortion coverage policies [Appendix B, Column (2)] was obtained from the Guttmacher Institute.[6]

Data analysis

Coverage for out-of-state abortion was summarized based on agency representatives' responses to the initial question "I was wondering if there's any way that the abortion would be covered by her ____[State Medicaid Agency]___ if she does it out of state?" and subsequent question "What if it's an emergency?" If agency representatives indicated no to the first question and yes to coverage in a medical emergency, coverage was summarized as 'Medical Emergency Covered.' If agency staff member answered yes to the first question or mentioned the possibility of coverage beyond medical emergency (rape and incest) during the call, coverage was summarized as 'Coverage beyond medical emergency.'

"I was wondering if there's any way that the abortion would be covered by her ____[State Medicaid Agency]___ if she does it out of state?" Summary of Response	"What if it's an emergency?" Summary of Response	Coded As
No	No	Not covered in medical emergency *
No, did not mention coverage circumstances beyond medical emergency, or did not know	Yes	Medical emergency covered
Yes or mentioned coverage circumstances including and beyond medical emergency in which abortion would be covered (i.e. rape, incest, with prior authorization)	-	Coverage beyond medical emergency
Did not know	Did not know	Did not know

*One state (Mississippi) representative indicated that rape and incest would be covered but abortion would not be covered if a pregnant person's life were in danger. This state was coded as not covered in a medical emergency.

© 2023 Khidir H et al. *JAMA Network Open.*

eReferences.

1. Dennis A, Blanchard K. A mystery caller evaluation of Medicaid staff responses about state coverage of abortion care. *Womens Health Issues Off Publ Jacobs Inst Womens Health*. 2012;22(2):e143-148. doi:10.1016/j.whi.2011.11.001

2. Dennis A, Blanchard K. Abortion providers' experiences with Medicaid abortion coverage policies: a qualitative multistate study. *Health Serv Res*. 2013;48(1):236-252. doi:10.1111/j.1475-6773.2012.01443.x

3. Dennis A, Manski R, Blanchard K. Does Medicaid Coverage Matter?: A Qualitative Multi-State Study of Abortion Affordability for Low-income Women. *J Health Care Poor Underserved*. 2014;25(4):1571-1585. doi:10.1353/hpu.2014.0151

4. Center for Reproductive Rights. After Roe Fell: Abortion Laws by State. Accessed September 11, 2023. https://web.archive.org/web/20230000000000*/https://reproductiverights.org/maps/abortion-laws-by-state/

5. Guttmacher Institute. Interactive Map: US Abortion Policies and Access After Roe. Accessed September 11, 2023. https://web.archive.org/web/20230000000000*/https://states.guttmacher.org/policies/

6. Guttmacher Institute. State Funding of Abortion Under Medicaid. Published August 2022. Accessed August 30, 2023. https://web.archive.org/web/20220803195047/https://www.guttmacher.org/state-policy/explore/state-funding-abortion-under-medicaid

© 2023 Khidir H et al. *JAMA Network Open.*

eTable. (1) Legal Status of Abortion and (2) State Medicaid Abortion Coverage Policies in Each State or District in the United States at the Time of Data Collection

State	(1) Legal Status of Abortion at time of data collection	(2) State Medicaid Coverage Policies for Abortion Care (Guttmacher 2022)
Alabama	Near total ban	Life Endangerment, rape and incest covered
Alaska	No restriction	All or most medically necessary abortions covered
Arizona	Banned after 15 weeks[a]	Life Endangerment, rape and incest covered
Arkansas	Near total ban	Life Endangerment, rape and incest covered
California	Banned at viability	All or most medically necessary abortions covered
Colorado	No restriction	Life Endangerment, rape and incest covered
Connecticut	Banned at viability	All or most medically necessary abortions covered
Delaware	Banned at viability	Life Endangerment, rape and incest covered
District of Columbia	Banned at viability	Life Endangerment, rape and incest covered
Florida	Banned at 15 weeks	Life Endangerment, rape and incest covered
Georgia	Banned at 6 weeks	Life Endangerment, rape and incest covered
Hawaii	Banned at viability	All or most medically necessary abortions covered
Idaho	Near total ban[a]	Life Endangerment, rape and incest covered
Illinois	Banned at viability	All or most medically necessary abortions covered
Indiana	Banned at 22 weeks[a]	Life Endangerment, rape and incest, and threat to physical health covered
Iowa	Banned at 22 weeks	Life Endangerment, rape and incest + fetal impairment covered
Kansas	Banned at 22 weeks	Life Endangerment, rape and incest covered
Kentucky	Near total ban[a]	Life Endangerment, rape and incest covered
Louisiana	Near total ban[a]	Life Endangerment, rape and incest covered
Maine	Banned at viability	All or most medically necessary abortions covered
Maryland	Viability[a]	All or most medically necessary abortions covered
Massachusetts	Banned at 24 weeks	All or most medically necessary abortions covered
Michigan	Banned at viability	Life Endangerment, rape and incest covered
Minnesota	No restriction[a]	All or most medically necessary abortions covered
Mississippi	Near total ban	Life Endangerment, rape and incest, and fetal impairment covered
Missouri	Near total ban	Life Endangerment, rape and incest covered
Montana	Banned at viability	All or most medically necessary abortions covered

© 2023 Khidir H et al. *JAMA Network Open.*

**Nebraska**	Banned at 22 weeks	Life Endangerment, rape and incest covered
**Nevada**	24 weeks or later[a]	Life Endangerment, rape and incest covered
**New Hampshire**	Banned at 24 week	Life Endangerment, rape and incest covered
**New Jersey**	No restriction	All or most medically necessary abortions covered
**New Mexico**	No restriction	All or most medically necessary abortions covered
**New York**	Banned at viability	All or most medically necessary abortions covered
**North Carolina**	Banned at 20 weeks[a]	Life Endangerment, rape and incest covered
**North Dakota**	Banned at 22 weeks	Life Endangerment, rape and incest covered
**Ohio**	Banned at 6 weeks[a]	Life Endangerment, rape and incest covered
**Oklahoma**	Near total ban	Life Endangerment, rape and incest covered
**Oregon**	No restriction	All or most medically necessary abortions covered
**Pennsylvania**	Banned at 24 weeks	Life Endangerment, rape and incest covered
**Rhode Island**	Banned at viability	Life Endangerment, rape and incest covered
**South Carolina**	Banned at 22 weeks[a]	Life Endangerment, rape and incest covered
**South Dakota**	Near total ban	Covers Life Endangerment Only covered
**Tennessee**	Near total ban*	Life Endangerment, rape and incest covered
**Texas**	Near total ban	Life Endangerment, rape and incest covered
**Utah**	Banned at 18 weeks	Life Endangerment, rape and incest, and threat to physical health covered
**Vermont**	No restriction	All or most medically necessary abortions covered
**Virginia**	Banned at 3rd trimester[a]	Life Endangerment, rape and incest and fetal impairment covered
**Washington**	Banned at viability	All or most medically necessary abortions covered
**West Virginia**	Banned at 22 weeks[a]	Life Endangerment, rape and incest, threat to physical health, and fetal impairment covered
**Wisconsin**	Banned at 22 weeks	Life Endangerment, rape and incest, and threat to physical health covered
**Wyoming**	Banned at 22 weeks[a]	Life Endangerment, rape and incest covered

[a]Guttmacher Institute and Center for Reproductive Rights websites reported different policies for the date of data collection, adjudicated using a third data source

© 2023 Khidir H et al. *JAMA Network Open.*
