# Supplementary material for: Mystery Shopper Study of State Medicaid Coverage for Out-of-State Abortion Care
Source: JAMA Netw Open. 2023 Nov 15;6(11):e2343569. doi: 10.1001/jamanetworkopen.2023.43569 (PMC10652153; doi:10.1001/jamanetworkopen.2023.43569)
Supplement: Supplement 2. — Data Sharing Statement [file jamanetwopen-e2343569-s002.pdf]

Data Sharing Statement

Khidir. Mystery Shopper Study of State Medicaid Coverage for Out-of-State Abortion Care. *JAMA Netw Open*. Published November 17, 2023. doi:10.1001/jamanetworkopen.2023.43569

Data
Data available: No

Additional Information
Explanation for why data not available: To protect the identities of Medicaid agency staff who were surveyed through mystery shopper calls, data will not be made available.
